# Study on plant protection unmanned aerial vehicle spraying technology based on the thrips population activity patterns during the cotton flowering period

**DOI:** 10.3389/fpls.2024.1337560

**Published:** 2024-06-26

**Authors:** Yapeng Liu, Zechen Dou, Hao Ren, Xiaolong Ma, Caiyue Liu, Muhammad Qasim, Xiaoqiang Han

**Affiliations:** Key Laboratory of Oasis Agricultural Pest Management and Plant Protection Utilization, College of Agriculture, Shihezi University, Shihezi, China

**Keywords:** cotton, thrips, flowering period, unmanned aerial vehicle, spraying technology

## Abstract

Over the years, thrips have transitioned from a minor nuisance to a major problem, significantly impacting the yield and quality of cotton. Unmanned aerial vehicles (UAVs) for plant protection have emerged as an effective alternative to traditional pesticide spraying equipment. UAVs offer advantages such as avoiding crop damage and enhancing pesticide deposition on the plants and have become the primary choice for pesticide application in cotton fields. In this study, a 2-year field experiment found that the thrips population in a cotton field in Xinjiang, China, exhibited gradual growth during the early flowering phase, peaking in late July. The thrips population gradually shifted from the lower canopy to the upper canopy as the cotton flowers opened layer by layer. From 09:00 to 11:00 (GMT+8) and 19:00 to 21:00 (GMT+8), thrips mainly flew outside the flowers, while from 17:00 to 19:00 (GMT+8), they mostly inhabited the inner whorls of flowers. The insecticides 10% cyantraniliprole oil dispersion and 10% spinetoram suspension concentrate, sprayed by UAV, had the best control effect on thrips, with 80.51% and 79.22% control effect after 7 days of spraying, respectively. The optimal spraying time for 10% cyantraniliprole oil dispersion was 19:00 (GMT+8), and the control effect on thrips reached 91.16% at 7 days of spraying. During the cotton flowering period, thrips inhabited flowers in the evening and flew outside during the day. The best control effect on thrips was achieved with UAV-sprayed 10% cyantraniliprole oil dispersion at 19:00 (GMT+8).

## 1 Introduction

Cotton is grown in more than 75 countries across the globe and is a significant source of fiber, oil, and several other products (Shahrajabian et al., 2020; Singh et al., 2023). Xinjiang is an important cotton production base in China and the world. In 2022, the planting area and cotton yield in Xinjiang accounted for 83.22% and 90.18% of the total in China, respectively (National Bureau of Statistics, 2022). While the cotton area and yield in Xinjiang are gradually increasing, diseases, pests, and weeds are also a constant threat to the safe production of cotton in the region. More than 300 pests harm cotton, with over 30 commonly occurring pests (Lu, 2021). In recent years, the occurrence of thrips (*Thrips tabaci* Lindeman and *Frankliniella intonsa* Trybom) in cotton fields has been increasing year by year, gradually evolving from a minor pest to a major pest in Xinjiang (Pan et al., 2018). Thrips damage the cotton epidermal tissue by sucking plant sap and cause “headless cotton” and “multi-headed cotton” during the cotton seedling stage, affecting the normal growth of cotton. Severe injury can even result in seedling death. In the later stages of cotton growth, thrips can harm cotton bolls, causing them to stiffen or crack, directly affecting the cotton yield (Chappell et al., 2020; Reitz et al., 2020; Wang, 2020). In 2020, the area under thrips attacks in Xinjiang was 215.4 ha, an increase of 57.2 ha compared with 2019, and the damage during the flowering and boll stage was much more severe than that during the seedling stage (Yan et al., 2021). In addition, the high-density farming method used in Xinjiang causes mechanical damage to cotton when pesticides are sprayed with a boom sprayer during the middle and later stages of cotton growth, significantly impacting cotton growth and production (Meng et al., 2019).

The use of pesticides remains the main method of controlling thrips. Organophosphorus, carbamate, pyrethroid, and neonicotinoid insecticides are the main insecticides used to control thrips. Different types of insecticides have different mechanisms of action, and their effectiveness in controlling thrips also varies to some extent. Due to the unreasonable application of pesticides, the resistance of thrips to insecticides has also developed rapidly (Huseth et al., 2016). Utilizing the predatory function of natural enemies has also become an important measure for controlling thrips. *Hippodamia variegata*, *Orius strigicollis*, *Erigonidium graminicolum*, *Propylea japonica*, and *Coccinella septempunctata* are important natural enemies of thrips (Deligeorgidis et al, 2010; Dang et al., 2023).

Unmanned aerial vehicles (UAVs) are used to spray pesticides on various crops, including wheat, rice, corn, fruit trees, tea, and cotton. This method can improve the deposition of pesticides on target crops, prevent mechanical damage to crops sprayed by boom sprayers, and considerably increase operating efficiency (Zhang et al., 2020). Thrips’ short developmental time, robust reproductive potential, frequent outbreaks, and extreme generation overlap make thrips prevention and management challenging. Chen et al. (2016) discovered that lotus thrips (*Scirtothrips dorsalis* Hood) could be successfully controlled by spraying avermectin using UAVs. Yuan et al. (2017) found that UAV spraying had better control effects on cowpea (*Vigna unguiculata* L.) thrips [*Megalurothrips usitatus* (Bagnall)] than manual spraying at a lower dosage. Lin et al. (2020) found that spraying insecticides using UAVs could achieve a control effect of 83.5% on sugarcane thrips; the thrips control effect was above 83.16% at the recommended dosage, and the required dosage was 25% less than that required for manual spraying (Liu et al., 2023). Fang et al. conducted field experiments to study the droplet density, coverage rate, deposition amount, droplet uniformity, and control effect on cotton thrips in the cotton canopy after spraying 25% thiamethoxam water-dispersible granules via UAV and identified the optimal parameters for using a UAV to control thrips during flowering in cotton fields (Fang et al., 2023).

In order to further improve the effectiveness of UAV spraying insecticides to control thrips, we conducted a 2-year experiment and conducted a detailed investigation of the daytime activity patterns of thrips. Based on thrips activity patterns, we further carried out screening of insecticides and timing of pesticide spraying and established a technical system for cotton field UAV spraying insecticides to control thrips. The aim of this study was to provide a scientific basis for effectively controlling thrips in cotton fields.

## 2 Materials and methods

### 2.1 Materials

Overall, nine formulations of different pesticides were collected from various companies to investigate their potential against cotton thrips. These included the following: 10% imidacloprid wettable powder (WP) (Guangdong Dafeng Plant Protection Technology Co., Ltd., Zhuhai, China), applied at 600 g/hm^2^; 600 g/L imidacloprid suspension concentrate (SC) (Shenzhen Noposion International Investment Co., Ltd., Shenzhen, China), applied at 100 mL/hm^2^; 70% imidacloprid water-dispersible granules (WG) (Shaanxi Huarong Kaiwei Biological Co., Ltd., Xi’an, China), applied at 85.7 g/hm^2^; 20% imidacloprid soluble concentrate (SL) (Shenzhen Noposion International Investment Co., Ltd., Shenzhen, China), applied at 300 mL/hm^2^; 25% thiamethoxam WG [Syngenta (Suzhou) Crop Protection Co., Ltd., Suzhou, China], applied at 225 g/hm^2^; 25 g/L deltamethrin emulsifiable concentrate (EC) (Jiangsu Huifeng Biological Agriculture Co., Ltd., Yancheng, China), applied at 600 mL/hm^2^; 1% emamectin benzoate micro-emulsion (ME) [Adama Huifeng (Jiangsu) Co., Ltd., Yancheng, China], applied at 360 mL/hm^2^; 10% spinetoram SC (Corteva Agriscience, Wilmington, DE, USA), applied at 600 mL/hm^2^; 10% cyantraniliprole oil dispersion (OD) (FMC, Philadelphia, PA, USA), applied at 600 mL/hm^2^; and Beidatong (Hebei Mingshun Agric. Technology Co., Ltd., Shijiazhuang, China), applied at 450 g/hm^2^.

The UAV (Dajiang T30) was supplied by Shenzhen Dajiang Innovation Technology Co., Ltd. (Shenzhen, China). The volume of the tank was 40 L, and the tank had dimensions of 2,858 mm × 2,685 mm × 790 mm (length × width × height) (arm deployment, paddle deployment), six rotors, and 16 SX11001VS nozzles. The operation parameters of the T30 UAV were input using the intelligent handheld terminal, and carrier phase difference technology was used for accurate flight positioning.

### 2.2 Field plots

This study was conducted at Beiquan town, Xinjiang Production and Construction Crops (44°20′7″N, 85°59′41″E), Shihezi, China, during 2022 and 2023. The field had been continuously planted with cotton for many years, and the ‘Huiyuan 720’ cotton variety was used in this research during both 2022 and 2023. The conventional wide and narrow row cotton sowing mode was adopted, with six rows of one film and 66 + 10 cm spacing. Drip irrigation with one film and two tubes was used throughout the entire growth period. The planting density of cotton was approximately 180,000 plants/hm^2^ and 185,000 plants/hm^2^ for 2022 and 2023, respectively.

### 2.3 Method

#### 2.3.1 Investigation of the population dynamics and activity rhythm of thrips during the cotton flowering period

In 2022, a thrips population dynamics survey was conducted during the cotton flowering period (July 1 to August 2). Surveying was performed every 2 days, with a fixed survey time from 17:00 to 19:00 (GMT+8). The number of thrips was investigated by five-point sampling, with 10 cotton flowers randomly selected from the survey area (100 m × 80 m). The thrips in the flowers were knocked down onto yellow sticky card traps, and the total number of thrips at each instar on the card traps was counted. In 2023, a repeated investigation was conducted during the peak period of thrips occurrence (July 23 to August 2).

**Figure 1:**
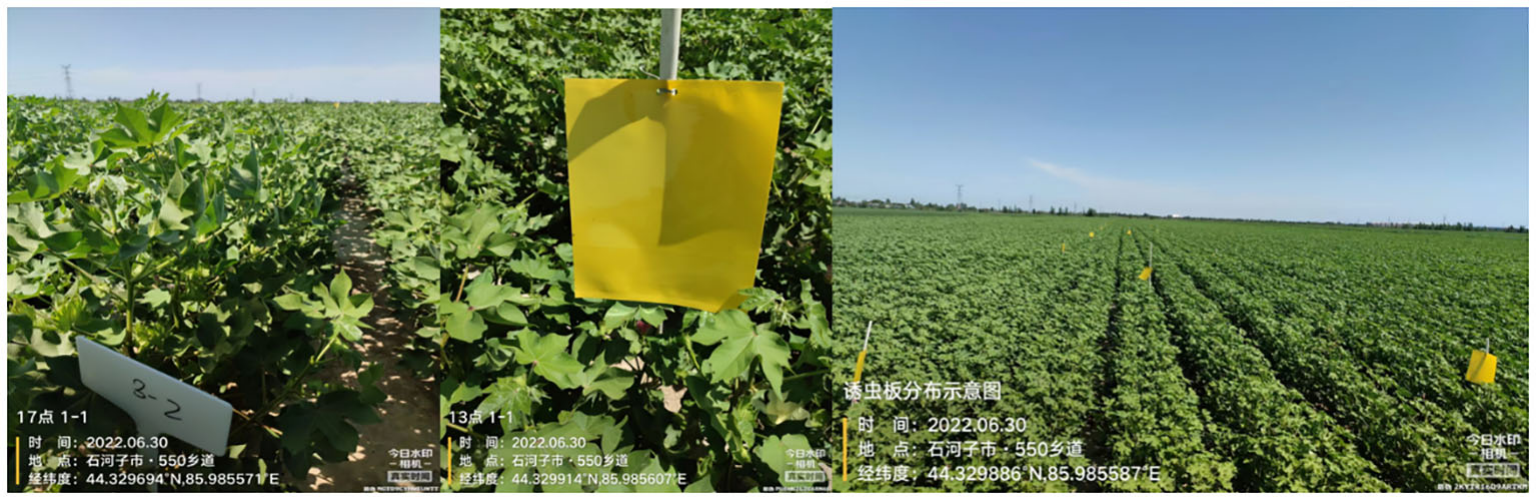
Investigation of the activity rhythm of thrips during the cotton flowering period.

**Figure 2:**
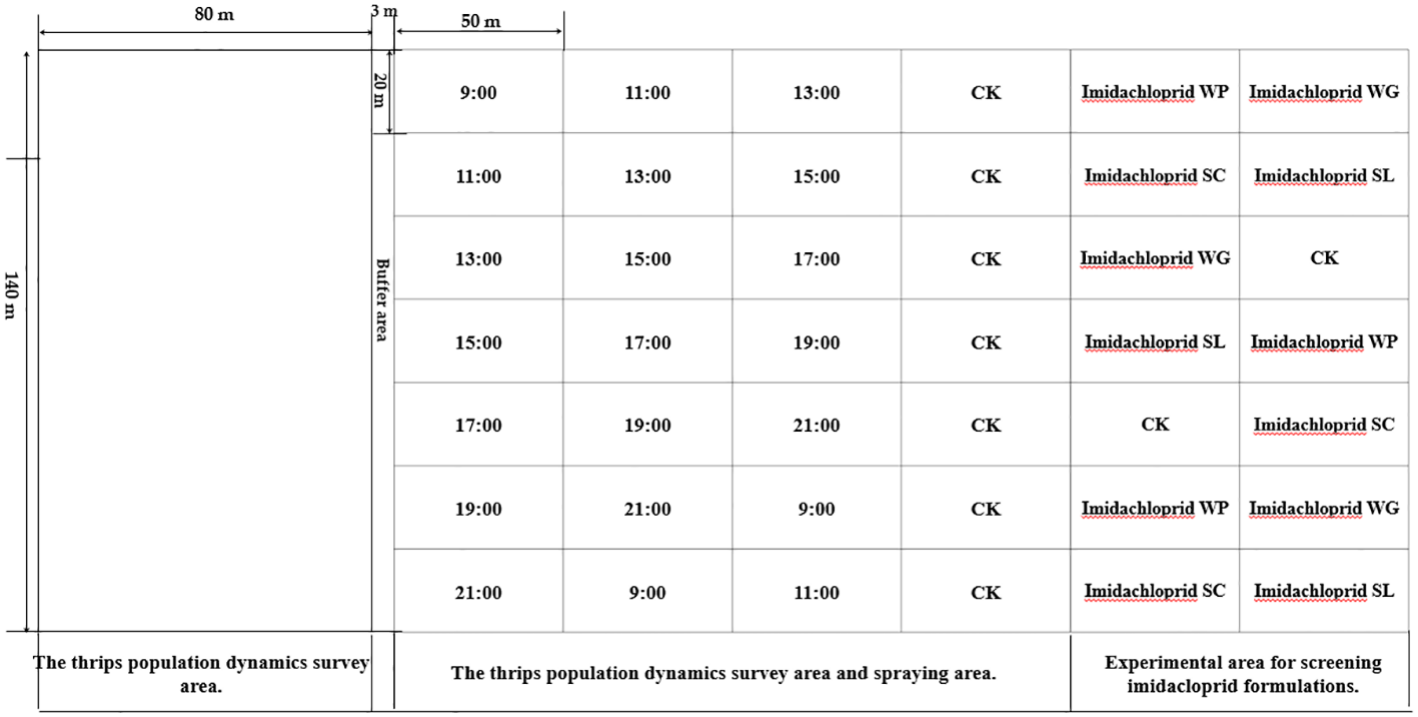
Experimental layout diagram.

On July 15, 2022, a survey was conducted on the activity rhythm of thrips during the cotton flowering period (with an average of over 1,000 thrips per 50 cotton plants). Surveying was performed every 2 days. In the survey area (20 m × 50 m), yellow sticky card traps (fixed in a 1.2-m-long plastic tube and placed at five points in the survey area) were used to investigate the number of active thrips outside the flowers (**Figures 1**, **2**). The number of thrips in the cotton flowers was investigated by five-point sampling, with 50 cotton flowers randomly selected from the same survey area, and surveying was repeated three times. Each survey was conducted every 2 h from 09:00 to 21:00 (GMT+8) and repeated three times. In 2023, a repeated investigation was conducted during the peak period of thrips occurrence (July 23 to August 2).

#### 2.3.2 Selection of insecticides and spraying timing for controlling thrips

On July 18, 2022, from 17:00 to 19:00 (GMT+8), a screening experiment was conducted to control thrips during cotton flowering. During the test, the route spacing of the T30 UAV was 5 m, the flight speed was 2 m/s, and the flight height was 2 m (from the top of the cotton canopy). The average wind speed during spraying was 1.7 m/s, the relative humidity was 53.2%, and the ambient temperature was 30.1°C–34.2°C (Kestrel 5500, Nielsen-Kellerman, Boothwyn, PA, USA). During the experiment, cotton was in its peak flowering period, with an average plant height of 1.12 m. This study first screened the formulation of imidacloprid, the main insecticide used to control thrips in cotton fields in Xinjiang. Due to the rapid development of resistance to neonicotinoid insecticides in thrips, screening was also conducted for new insecticides to control thrips. There were 10 treatments, with three replicates for each treatment (20 m × 50 m) (**Table 1**; **Figure 2**). The insect population was investigated before spraying, and the number of residual insects was investigated on 1 day, 3 days, and 7 days after spraying. The values of the control effect were then calculated according to Equations 1, 2:

**Table 1:** Selection of spraying timing for thrips control (2022).

Treatment	Spraying time	Wind speed (m/s)	Temperature (°C)	Relative humidity (%)
1	09:00	0.3	26.1	54.6
2	11:00	0.7	29.6	52.3
3	13:00	1.3	32.7	42.8
4	15:00	1.2	33.9	41.1
5	17:00	1.5	30.1	38.6
6	19:00	0.9	29.4	39.6
7	21:00	0.4	27.2	46.3
8	CK	–	–	–


(1) $$\begin{array}{c} \text{Reduction rate of insect population }\left(\%\right)\\ \;=\frac{\text{Insect population before spraying}-\text{Insect population after spraying}}{\text{Insect population before spraying }}\times 100, \end{array}$$

(2) $$\text{Control effect }\left(\%\right)=\frac{\text{Reduction rate of insect population in the treatment}-\text{Reduction rate of insect population in the CK}}{100-\text{Reduction rate of insect population in CK}}\times 100.$$

On July 25, 2022, and August 2, 2023, a screening experiment was conducted regarding the spraying timing for thrips control during the cotton flowering period. The pesticide was sprayed every 2 h from 09:00 to 21:00 (GMT+8). The 10% cyantraniliprole OD, which had the best control effect on thrips, was selected as the test insecticide at a dosage of 600 mL/hm^2^ and added to Beidatong at a dosage of 300 mL/hm^2^. The UAV spraying parameters were the same as before. The meteorological information for each application time is shown in **Table 1**. On August 2, 2023, with 10% cyantraniliprole OD as the test insecticide, a UAV was used at 19:00 (GMT+8), when thrips were the most likely to inhabit flowers, to further verify the impact of spraying time on the thrips control effect (**Table 1**).

### 2.4 Data statistics and processing

Data were compared across different application rates using analysis of variance (ANOVA). The confidence interval was set to 95% and *p* < 0.05, and 99% and *p* < 0.01 were chosen to indicate a significant difference between the two groups. SPSS 18.0 was used for data processing and analysis, and the figures were designed using SigmaPlot 12.5 and Origin 2021.

## 3 Results

### 3.1 Thrips population dynamics during the cotton flowering period

**Figure 3:**
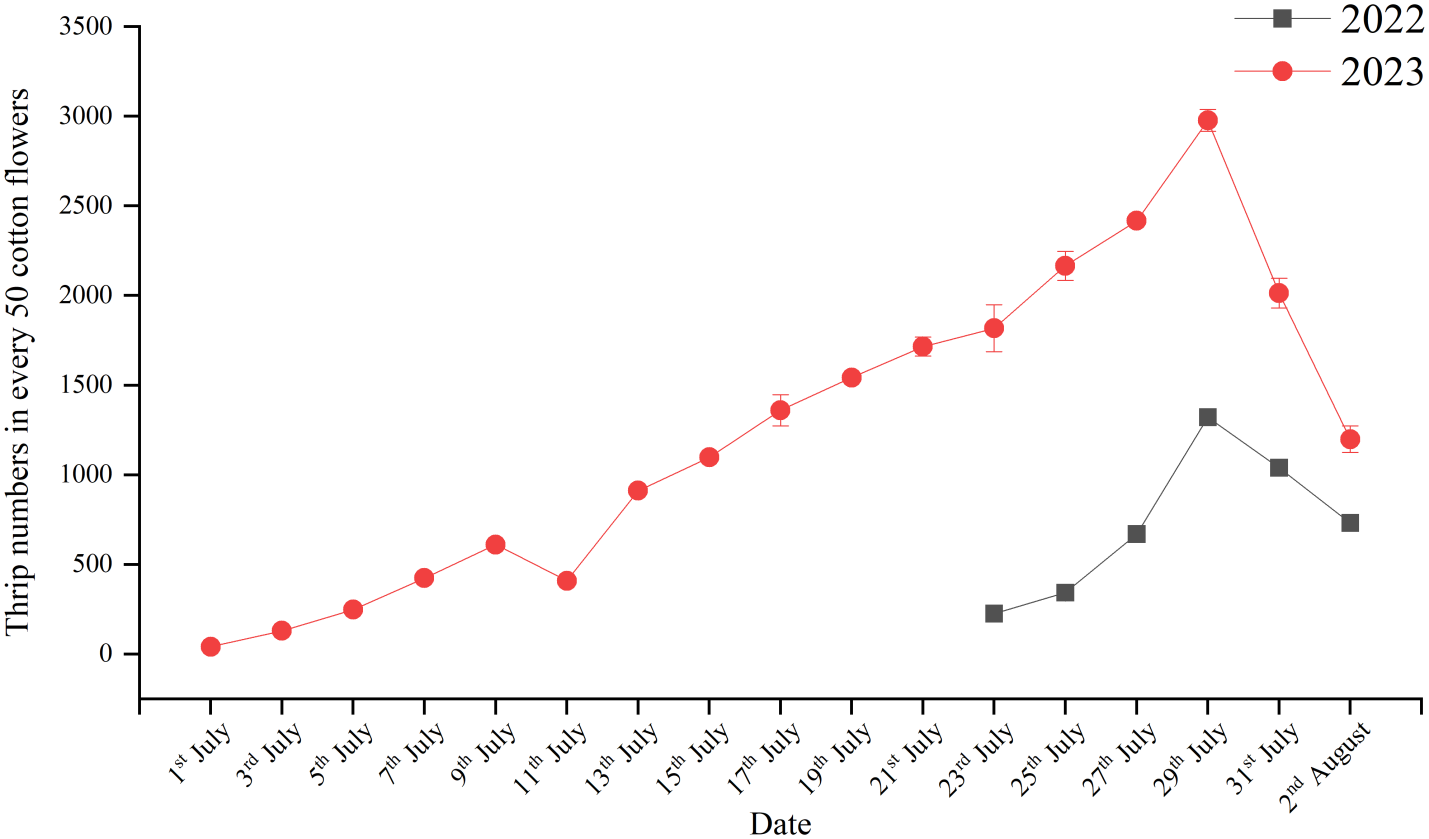
Thrips population dynamics during the cotton flowering period.

The results of the investigation on the population dynamics of thrips during cotton flowering in 2022 are shown in **Figure 3**. The population of thrips within cotton flowers was consistent with the cotton flowering period. In the initial stage of cotton flowering (July 1–15), fewer flowers were present, and the number of thrips inside the flowers increased slowly (affected by rainfall on July 11, the number of thrips decreased compared with the previous survey). During the peak flowering period of cotton (after July 15), the number of thrips in the flowers reached 549 (in 50 flowers). Afterward, under continuous high temperatures (above 30°C), the number of thrips in cotton flowers rapidly increased and reached the peak on July 29, with an average of 2,977 (in 50 flowers). At the end of the cotton flowering period, the flowers decayed and fell, and the number of thrips in the flowers rapidly decreased. The average number of thrips surveyed on July 31 and August 2 was 2013 and 1198 (in 50 flowers), respectively. Overall, the thrips population during cotton flowering in Xinjiang showed an exponential growth trend. The number of thrips in 2023 was significantly lower than that in 2022, but the population dynamics were consistent with those in 2022.

**Figure 4:**
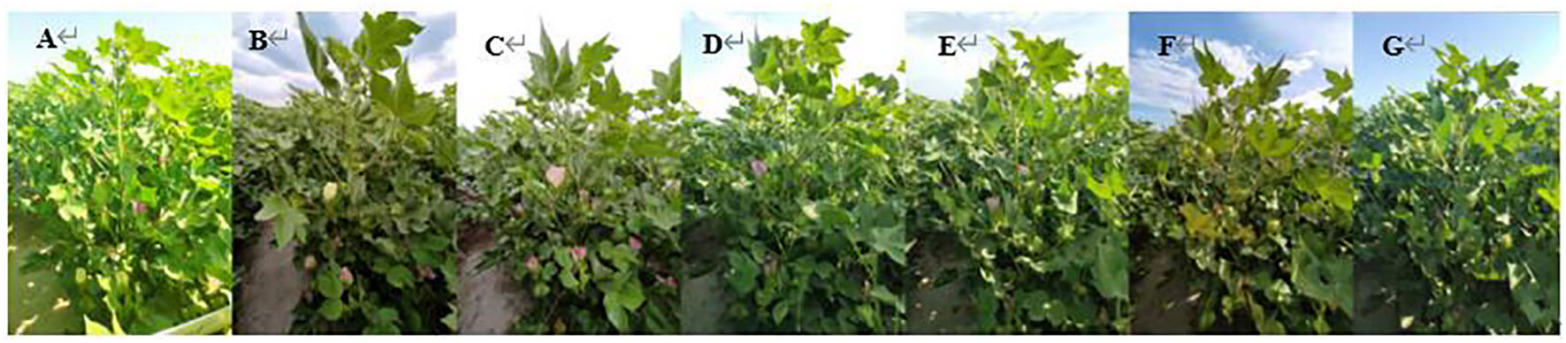
Cotton flowers bloom successively from the lower canopy to the upper canopy. The photographs from left to right show the same cotton plant on July 7 **(A)**, July 9 **(B)**, July 11 **(C)**, July 13 **(D)**, July 15 **(E)**, July 17 **(F)**, and July 19, 200 **(G)**.

This study also investigated the distribution of intra-floral thrips in different cotton canopies. The results indicated that the distribution of thrips in the cotton canopy was closely related to the flowering pattern of cotton flowers (**Figure 4**). At the beginning of cotton flowering, the number of fruit branches in cotton plants ranges from 10 to 13, with approximately two to three buds on each branch. Cotton flowers gradually bloom from the first fruit branch at the bottom of the plant, with flowers opening progressively from the lower canopy to the upper canopy. The flowering time of cotton flowers is approximately 5 days, with the lower canopy flowers blooming first, while the middle and upper canopies remain in bud. At this time, thrips were mainly distributed in the lower canopy in this study. At the peak flowering stage, the flowers in the middle and upper canopy were in full bloom, but at this time, the flowers in the lower canopy had withered and bolled, and the distribution of the thrips population had shifted from the lower canopy to the upper canopy.

### 3.2 Diurnal activity patterns of thrips during the cotton flowering period

**Figure 5:**
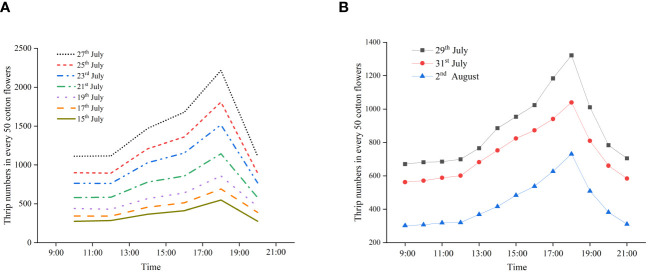
Diurnal activity patterns of thrips in flowers during the cotton flowering period in 2022 **(A)** and 2023 **(B)**.

The diurnal activity patterns of thrips in the flowers during the cotton flowering period are shown in **Figure 5**. According to the 2022 survey data, relatively few thrips inhabited or fed on flowers from 09:00 to 11:00 (GMT+8), and the average number of thrips was 631 (in 50 flowers). There was no significant change in the number of thrips in the flowers at 11:00–13:00 (GMT+8) compared with the previous period. At 13:00–15:00 (GMT+8) and 15:00–17:00 (GMT+8), the number of thrips in the flowers gradually increased to an average of 849 and 945, respectively (in 50 flowers). At 17:00–19:00 (GMT+8), the number of thrips in cotton flowers reached its maximum, with an average of 1,255 (in 50 flowers). At 19:00–21:00, the number of thrips in the flowers significantly decreased again, reaching an average of 640 (in 50 flowers) (**Figure 5A**). Although the number of thrips in each survey increased compared with the previous survey, the trend of changes in different periods was consistent. The results of the 2023 survey data were similar to those of 2022 (**Figure 5B**). Overall, there were relatively few thrips in the flowers at 09:00–13:00 (GMT+8), and the number of thrips significantly increased at 13:00–17:00 (GMT+8), reached the peak when the flowers bloomed at 17:00–19:00 (GMT+8), and rapidly decreased after the cotton flowers closed at 19:00 (GMT+8).

**Figure 6:**
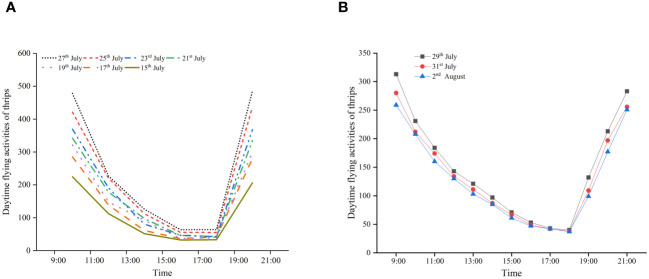
Diurnal activity patterns of thrips outside of flowers during the cotton flowering period in 2022 **(A)** and 2023 **(B)**.

The trend of changes in the number of flying thrips was opposite to the number of thrips in flowers during the cotton flowering period (**Figure 6**). At 09:00–11:00 (GMT+8) and 19:00–21:00 (GMT+8), the number of thrips was 349 and 345, respectively. At 11:00–13:00 (GMT+8), 13:00–15:00 (GMT+8), and 15:00–17:00 (GMT+8), the number of thrips gradually decreased to 173, 89, and 45, respectively. There was no significant change in the number of thrips at 17:00–19:00 (GMT+8) and 15:00–17:00 (GMT+8) (**Figure 6A**). In the seven surveys conducted in 2022, the trend of changes in the number of flying thrips during different periods was also consistent. The results of the 2023 survey were similar to those of 2022 (**Figure 6B**). Overall, thrips mainly flew outside the flowers at 09:00–13:00 (GMT+8), and their flight activity gradually weakened at 13:00–15:00 (GMT+8). There was almost no flight activity among thrips at 15:00–19:00 (GMT+8). The thrips flight activity revived at 19:00–21:00 (GMT+8). This pattern is closely linked to the blooming and closing of cotton flowers.

### 3.3 Selection of insecticides for controlling thrips

**Figure 7:**
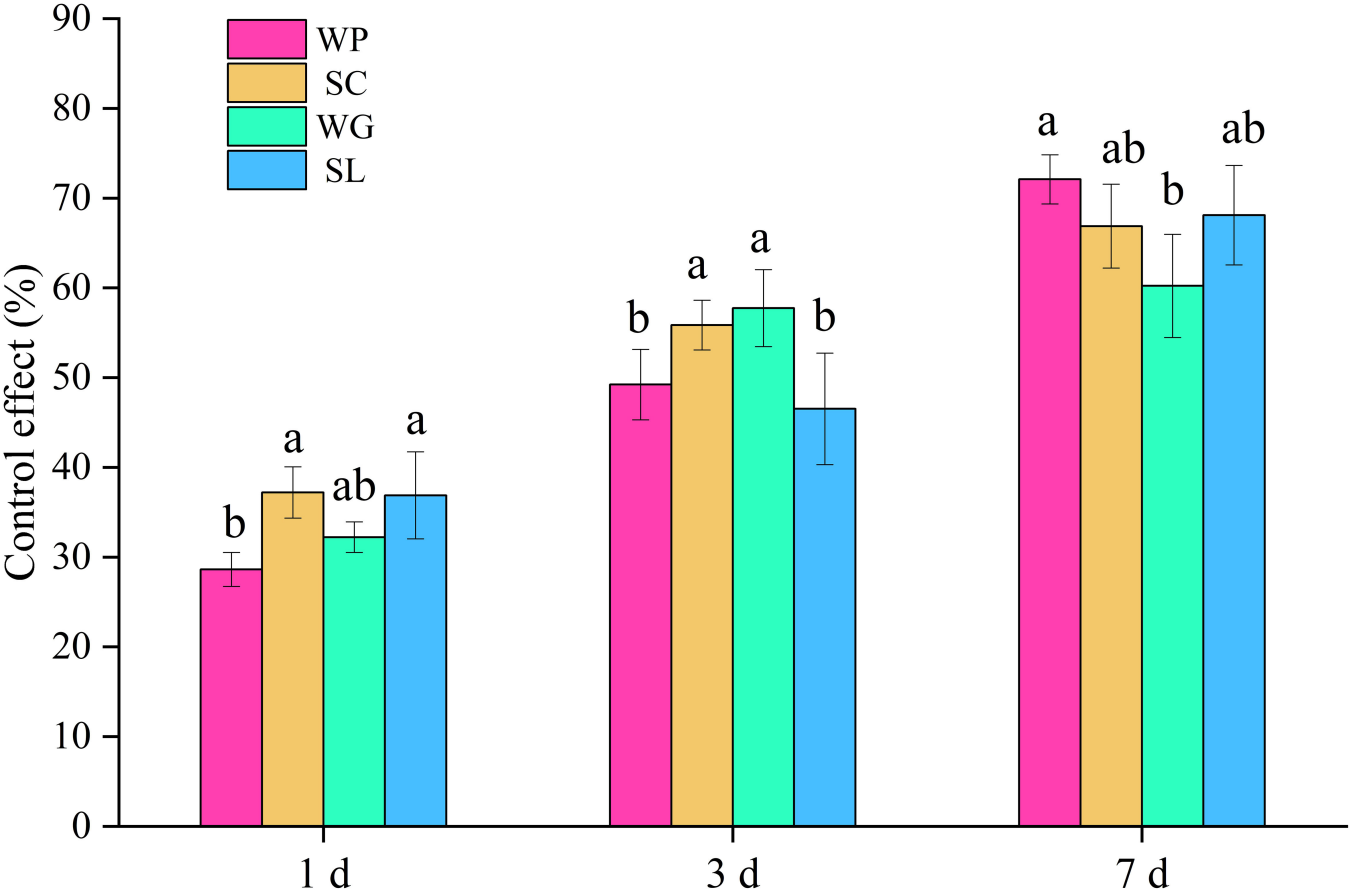
Effects of different imidacloprid formulations on the control of thrips in 2022. WP, 10% imidacloprid wettable powder; SC, 600 g/L imidacloprid suspension concentrate; WG, 70% imidacloprid water-dispersible granules; SL, 20% imidacloprid soluble concentrate.

Imidacloprid is the main insecticide used to control thrips in the cotton fields in Xinjiang, but the imidacloprid formulations on the market are relatively complex, which significantly affects the control effect after spraying via UAV. The control effects of different imidacloprid formulations on thrips during the cotton flowering period are shown in **Figure 7**. After 1 day of spraying (DOS), the 600 g/L imidacloprid SC and 20% imidacloprid SL showed better control effects at 37.21% and 36.88%, respectively, which were significantly higher than the control effects of 10% imidacloprid WP (28.63%). After 3 DOS, 70% imidacloprid WG had the best control effect, reaching 57.74%. This pesticide was followed by 600 g/L imidacloprid SC, with a control effect of 55.86%. The 10% imidacloprid WP and 20% imidacloprid SL had poor control effects at 49.23% and 46.53%, respectively. After 7 DOS, the highest to lowest control effects were 10% imidacloprid WP, 20% imidacloprid SL, 600 g/L imidacloprid SC, and 70% imidacloprid WG at 72.10%, 68.09%, 66.88%, and 60.22%, respectively. The above research results indicated that the formulations had an impact on the effective duration of imidacloprid against thrips.

**Figure 8:**
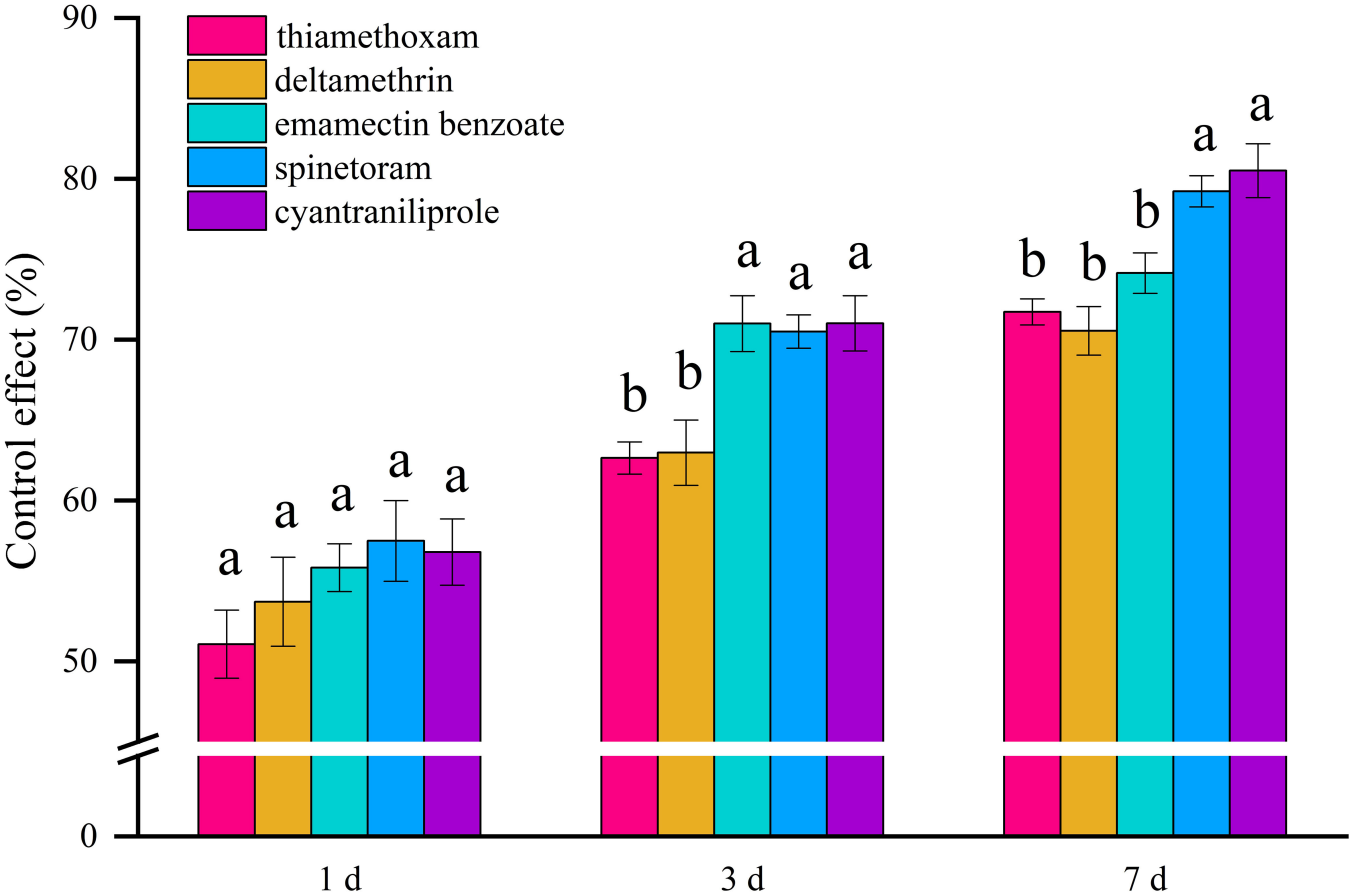
Control efficacy of five insecticides applied by unmanned aerial vehicle (UAV) spraying on thrips in a cotton field in 2022.

However, thrips have rapidly developed resistance to imidacloprid, making it necessary to screen new highly effective insecticides against thrips (Huseth et al., 2016). Thiamethoxam, deltamethrin, emamectin benzoate, spinetoram, and cyantraniliprole can all be used for the control of thrips, but the differences in their control effects on thrips during the cotton flowering period are not clear. The control effects of the above five insecticides on thrips during the cotton flowering period were studied when sprayed by UAV (**Figure 8**). At 1 DOS, the control effects of the five insecticides on thrips all exceeded 50%, but there was no significant difference between them. At 3 DOS, the control effects of 1% emamectin benzoate ME, 10% spinetoram SC, and 10% cyantraniliprole OD on thrips were 71.00%, 70.51%, and 71.02%, respectively, significantly higher than those of 25% thiamethoxam WG and 25 g/L deltamethrin EC (62.64% and 62.98%, respectively). At 7 DOS, the 10% cyantraniliprole OD and 10% spinetoram SC had the best control effect on thrips, with 80.51% and 79.22%, respectively.

### 3.4 Selection of spraying timing for thrips control

**Figure 9:**
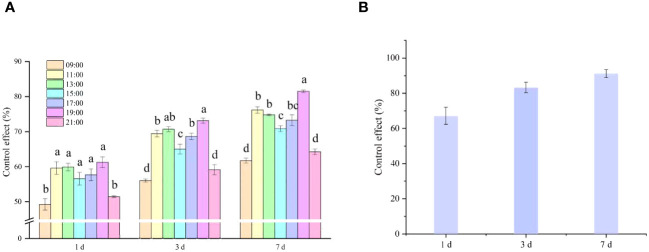
Control efficacy of 10% cyantraniliprole oil dispersion (OD) sprayed by unmanned aerial vehicle (UAV) on thrips in cotton fields in 2022 **(A)** and 2023 **(B)**.

After clarifying the activity patterns of thrips, 10% cyantraniliprole OD was selected as the test insecticide, and the effect of spraying timing on the thrips control effect was investigated (**Figure 9**). As shown in **Figure 9A**, after 1 DOS, the control effects on thrips when sprayed by UAV at 09:00 (GMT+8) and 21:00 (GMT+8) were 49.25% and 51.48%, respectively, significantly lower than the control effects of other treatments. After 3 DOS, the control effects on thrips sprayed by UAV at 19:00 and 13:00 were better at 73.14% and 70.70%, respectively, significantly higher than the control effects of other treatments. The control effects on thrips sprayed by UAV at 09:00 (GMT+8) and 21:00 (GMT+8) were the worst at 56.05% and 59.16%, respectively, significantly lower than the control effects of other treatments. After 7 DOS, the highest control effect on thrips was achieved when spraying was conducted at 19:00 (GMT+8) (81.51%), which was significantly better than the other spraying times. The control effects of spraying at 09:00 (GMT+8) and 21:00 (GMT+8) were 64.26% and 61.73%, respectively, significantly lower than the control effects of other spraying times. The results of the diurnal activity patterns of thrips indicated that at 19:00 (GMT+8), thrips mainly inhabited cotton flowers. At this time, thrips did not fly, and insecticides could directly come into contact with thrips. In 2023, 10% cyantraniliprole OD was sprayed via UAV at 19:00 (GMT+8), when the highest number of thrips were found in the flowers, and the control effect on thrips reached 91.16% after 7 DOS, showing an excellent control effect (**Figure 9B**).

## 4 Discussion

### 4.1 The daytime activity pattern of thrips

The spatial distribution of insect populations includes the following two aspects: the horizontal spatial distribution and the vertical spatial distribution. Insect migration and flight are often related to feeding and reproduction. During the cotton flowering period, the population of thrips gradually shifts from the lower canopy to the upper canopy as the cotton flowers bloom layer by layer from the bottom branches to the top of the plant. Previous studies showed that the daytime activities of thrips during the flowering periods of other field crops also exhibited certain patterns. Cho et al. (2000) found that during the flowering period of tomatoes, the number of *Frankliniella occidentalis* (Pergande) in the morning was significantly higher than that in the afternoon. Yan et al. (2017) found that the peak activity periods of *M. usitatus* (Bagnall) in a cowpea field in Hainan were from 08:00 to 10:00 (GMT+8) and 20:00 to 06:00 of the next day (GMT+8), they were almost inactive. Liang et al. (2010) found that during the flowering period of cucumbers in greenhouses, the number of flying *F. occidentalis* (Pergande) was the highest at 08:00–10:00 (GMT+8), slightly increased at 14:00–16:00 (GMT+8), and dropped to a very low level at 18:00 (GMT+8). Aliakbarpour and Salmah Md Rawi (2020) found that yellow sticky card traps captured the most thrips at 08:00–10:00 (GMT+8) and 14:00–16:00 (GMT+8) in a mango orchard in Malaysia, which represent the peak periods of thrips flight activity. The present study found that the thrips flew frequently at 09:00–11:00 (GMT+8) and 19:00–21:00 (GMT+8) every day, while they did not fly at 17:00–19:00 (GMT+8).

Obtaining a full understanding of the activity habits of insects has important reference value for developing effective pest management strategies. Thrips are flower-dwelling species that are often attracted to the aroma and color of flowers. Cotton flowers bloom in the morning and evening, luring thrips to feed. During this time, thrips are active and exhibit flight activities. The relative humidity in the field also has an impact on the flight of thrips. Liang (2010) found that there were very few flying thrips when the relative humidity of the environment was below 30% and above 90%, and the number of flying adult thrips was the highest when the relative humidity was 70%. Previous studies have also found that thunderstorms can cause thrips to fly in advance, possibly due to rapid changes in the atmospheric potential, temperature, or light, but there is no experimental evidence to confirm this possibility (Kirk, 2004). Terry and Gardner (1990) discovered in the Amazon that male *F. occidentalis* (Pergande) infesting cotton and alfalfa fields flock together on the surface of flowers or white plastic cups before a storm arrives. The present investigation showed that on cloudy and rainy days, the number of thrips inhabiting the flowers decreased, and the number of flights outside the flowers increased.

### 4.2 UAV spraying technology for controlling thrips

Thiamethoxam, deltamethrin, emamectin benzoate, spinetoram, and cyantraniliprole target the nicotinic acetylcholine receptors, sodium ionomer channel, glutamate-gated chloride ion channel, nicotinic acetylcholine receptors, and ryanodine receptor, respectively. These five insecticides have different mechanisms, and their control effects on thrips vary to some extent. This work found that 10% cyantraniliprole OD and 10% spinetoram SC displayed good control effects on thrips during the flowering period in cotton fields. The timing of pesticide spraying is closely related to the weather, crop growth status, and insect habits and significantly impacts the effectiveness of pest control (Meng et al., 2023). This study investigated the effect of the spraying time on the thrips control effect in cotton fields based on the daytime activity patterns of thrips. The results showed that spraying timing had a significant impact on the thrips control effect during the flowering period. The best time to conduct UAV spraying for the control of thrips during the cotton flowering period was 19:00 (GMT+8), followed by 11:00 (GMT+8). Spraying at 09:00 (GMT+8) and 21:00 (GMT+8) had the worst control effects. Thrips flew frequently in the morning and evening, and their contact probability with insecticide was reduced, resulting in poor efficacy. At 19:00 (GMT+8), thrips mainly inhabited the cotton flowers, and the probability of contact between insecticide and thrips increased, increasing the effectiveness of the insecticide. Although there were many thrips in cotton flowers at 13:00–17:00 (GMT+8), the loss and evaporation of UAV spraying were serious due to the high temperature and sunlight, which affected the contact between insecticide and thrips. Therefore, using UAVs to spray insecticides to control thrips during the cotton flowering period at 19:00 (GMT+8) in Xinjiang is recommended.

Mastering the activity patterns of pests, especially the inactive time of flying insects, is of great significance for their efficient control. In this study, a 2-year survey was conducted to clarify the activity patterns of thrips during the flowering period of cotton in Xinjiang. Using UAVs to spray pesticides during the inactive flight time of thrips can achieve efficient control of thrips. This study provides insights into thrips management during the flowering period in cotton fields.

## 5 Conclusion

Research conducted over two consecutive years revealed that the thrips population in cotton fields increased slowly during the early flowering stage and peaked at the end of July. As the cotton blossoms open layer by layer from the bottom to the top of the plant, the thrips population steadily moves from the lower canopy to the upper canopy. Thrips primarily flew outside the flowers between 09:00 and 11:00 (GMT+8) and 19:00 and 21:00 (GMT+8), and they primarily resided inside the blooms between 17:00 and 19:00 (GMT+8). The 10% spinetoram SC and 10% cyantraniliprole OD sprayed by the UAV had the best effects on thrips with control effects of 80.51% and 79.22% at 7 DOS, respectively. The best time to spray 10% cyantraniliprole OD on UAVs to control thrips was 19:00 (GMT+8), and at 7 DOS, the control impact on thrips was 91.16%. Thrips spend the night inside the blossoms and spend the day flying outdoors during the cotton blossoming season. When 10% cyantraniliprole OD was sprayed by the UAV at 19:00 (GMT+8) in Xinjiang, it showed the best control effect on thrips. The findings of this study provide a reference basis for developing more effective thrips control strategies in the cotton-growing region of Xinjiang. However, in this study, we mainly considered factors such as the activity pattern of thrips and the timing of pesticide spraying. In the future, we should combine meteorological conditions to carry out prediction and prediction of thrips, use remote sensing technology to identify and diagnose the harm of thrips, and establish a prescription map for the prevention and control of thrips, achieving precise control of cotton thrips.

## Data availability statement

The original contributions presented in the study are included in the article/supplementary material. Further inquiries can be directed to the corresponding author.

## Author contributions

YL: Data curation, Investigation, Methodology, Software, Visualization, Writing – original draft. ZD: Data curation, Investigation, Methodology, Writing – original draft. HR: Data curation, Investigation, Writing – original draft. XM: Investigation, Writing – original draft. CL: Software, Visualization, Writing – original draft. MQ: Supervision, Writing – review & editing. XH: Conceptualization, Funding acquisition, Methodology, Resources, Supervision, Writing – original draft, Writing – review & editing.
